# A case report of ‘Two-Hit’ digenic mutations in PAH: role of PADN in management

**DOI:** 10.3389/fphar.2025.1601777

**Published:** 2025-07-15

**Authors:** Zai-qiang Zhang, Zhou-qiang Qin, Sheng-kui Zhu, Yu-hong Zuo, Jia-wang Ding

**Affiliations:** ^1^ Department of Cardiology, The First College of Clinical Medical Sciences, China Three Gorges University, Yichang, Hubei, China; ^2^ Institute of Cardiovascular Diseases, China Three Gorges University, Yichang, Hubei, China; ^3^ Changyang Tujia Autonomous County People’s Hospital, Yichang, Hubei, China

**Keywords:** pulmonary arterial hypertension, digenic mutations, genetic pathogenesis, heart failure, case report

## Abstract

**Background:**

Pulmonary arterial hypertension (PAH) is a severe cardiopulmonary disorder characterized by progressive elevation of pulmonary vascular resistance, resulting to right ventricular dysfunction and premature mortality. Although genetic mutations are increasingly recognized in PAH pathogenesis, cases involving digenic mutations remain exceptionally rare.

**Case presentation:**

We report the case of a 47-year-old female presenting with a 5-year history of exertional dyspnea, which progressively worsened over the preceding 2 months. Diagnostic imaging revealed pulmonary artery dilatation and right heart enlargement, and right heart catheterization confirmed PAH with a mean pulmonary arterial pressure of 43 mmHg. Whole exome sequencing identified a novel heterozygous mutation in *FLNA* (c.4754C>T, p.Thr1585Met) and a known heterozygous mutation in *MMACHC* (c.609G>A, p.Trp203Ter). The patient was initiated on PAH-specific therapy and pulmonary artery denervation (PADN) treatment. Over a 2-year follow-up period, her symptoms significantly improved, with no evidence of heart failure progression.

**Conclusion:**

This case highlights a rare instance of PAH associated with digenic mutations in *FLNA* and *MMACHC*. The patient demonstrated a favorable response to targeted PAH therapy and PADN treatment, highlighting the importance of genetic screening and personalized treatment strategies in PAH management.

## Background

Pulmonary arterial hypertension (PAH) is a progressive and life-threatening disorder defined by a mean pulmonary artery pressure (mPAP) exceeding 20 mmHg at rest, accompanied by elevated pulmonary vascular resistance and right ventricular dysfunction (Humbert et al., 2023). The disease is driven by pathological remodeling of the pulmonary vasculature, ultimately leading to right heart failure and premature death. While the etiology of PAH is multifactorial, involving genetic, inflammatory, metabolic, and hemodynamic factors, the role of genetic mutations has garnered increasing attention. Mutations in genes such as *BMPR2*, *ALK1*, and *ENG* are well-documented in heritable PAH (Cuthbertson et al., 2023; Welch et al., 2023; Al Tabosh et al., 2024). However, cases involving digenic mutations are exceedingly rare. Here, we present a unique case of PAH associated with a novel heterozygous mutation in *FLNA* and a known heterozygous mutation in *MMACHC*. We observed that PAH-targeted therapies and pulmonary artery denervation (PADN) treatment were well tolerated by our patient and did not lead to heart failure during a 2-year follow-up period.

## Case presentation

A 47-year-old female was referred to our center with a 5-year history of progressive exertional dyspnea, which had significantly worsened over the preceding 2 months. The patient denied any family history of pulmonary hypertension or cardiopulmonary diseases. Her medical history included intellectual disability, schizophrenia, epilepsy, dysaudia, and hypothyroidism. Her father passed away due to an illness in his early years, with the exact cause remaining unknown. She was a teetotaler, nonsmoker and had no history of addictive drug use or other PH-associated risk factors. Her preliminary examination revealed the following physical examination parameters: blood pressure of 103/70 mmHg, heart rate of 71 bpm, respiratory rate of 19 breaths/min, oxygen saturation of 96% on room air, and New York Heart Association functional class III heart failure. Cardiac auscultation revealed an accentuated second pulmonic sound and diminished breath sounds bilaterally. Mild pitting edema was noted in the lower extremities. The laboratory examination results were as follows: cardiac troponin I (cTn I) 145 pg/mL, D-dimer 642 ng/mL, and NT-proBNP 3,026 pg/mL. Other laboratory values, such as erythrocyte sedimentation rate, HIV, C reactive protein, rheumatic factor, antinuclear antibody, antideoxyribonuclease, complements, lipoprotein A, factors II (prothrombin), VII, VIII, IX, XI, and XII, protein C, protein S, antiphos-pholipid antibody antibodies, antiphospholipid antibody, and anti-vasculitis antibody, were within the normal range.

**FIGURE 1:**
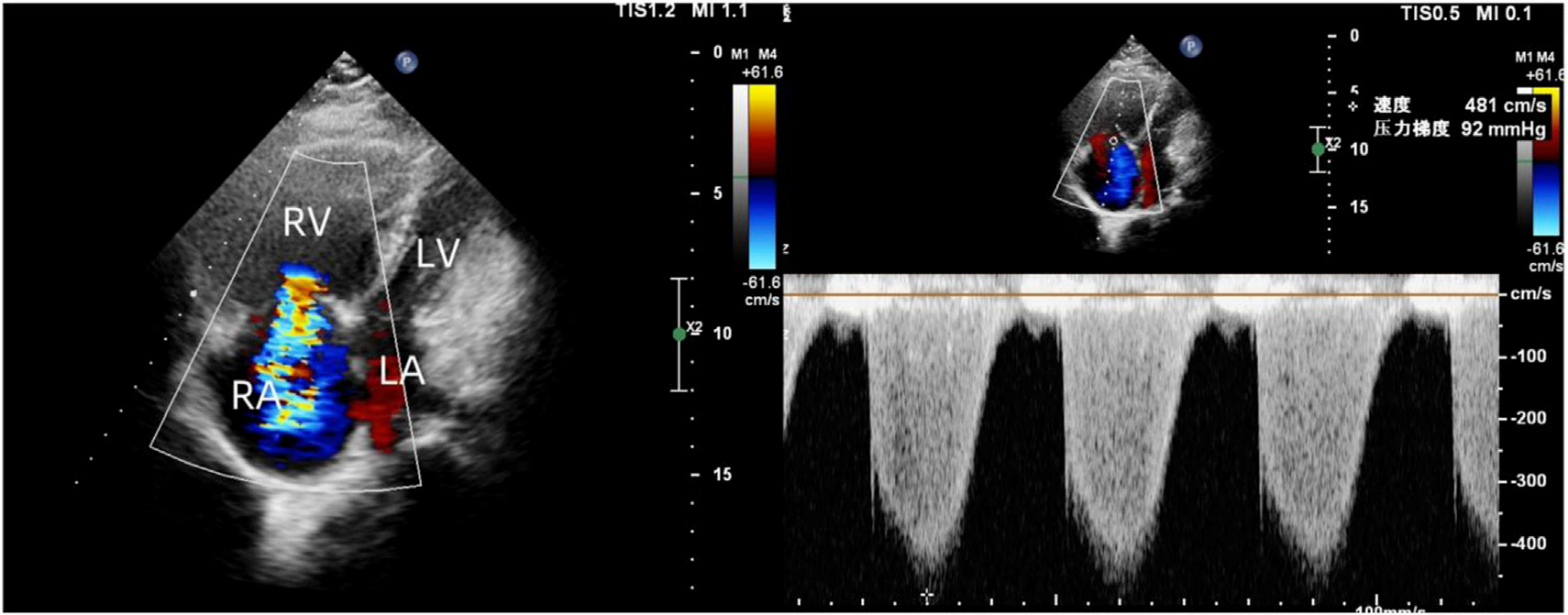
Transthoracic echocardiography revealed enlarged right heart, reduced right ventricular wall motion, and severe tricuspid regurgitation.

**FIGURE 2:**
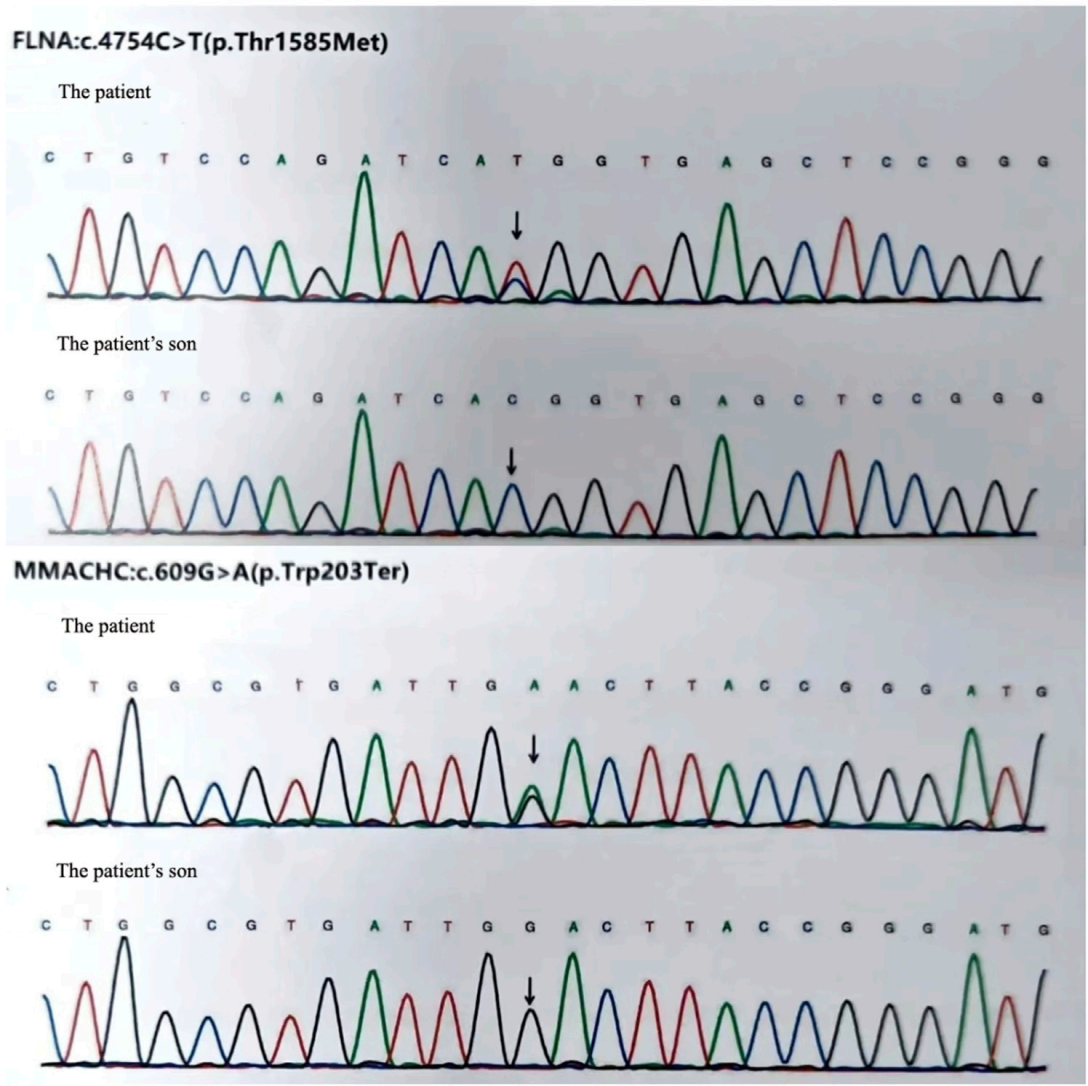
We identified a novel heterozygous mutation in *FLNA* (c.4754C>T, p.Thr1585Met) and a known heterozygous mutation in *MMACHC* (c.609G>A, p.Trp203Ter) in our patient. But the patient’s son revealed no evidence of these mutations.

**FIGURE 3:**
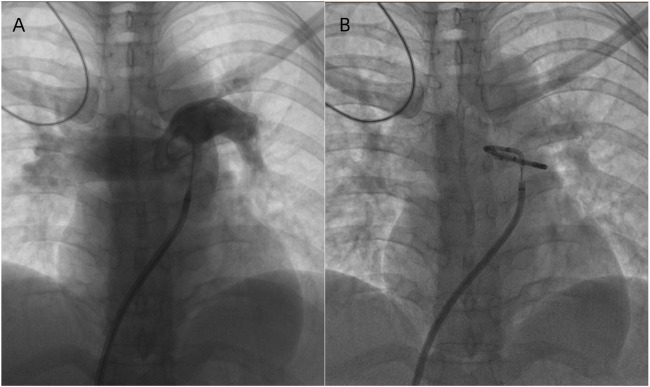
**(A)** Pulmonary arterial angiography and Identifying the ablation sites. **(B)** PA ablation is performed in the periconjunctional area between the distal main trunk and the ostial left branch.

To exclude pulmonary embolism, we performed cardiac MR image (MRI) and pulmonary ventilation perfusion imaging, and revealed pulmonary artery dilatation and right heart enlargement, which may indicate pulmonary arterial hypertension. Transthoracic echocardiography confirmed severe pulmonary hypertension, with an estimated pulmonary artery systolic pressure of 102 mmHg, preserved LVEF (62%), reduced right ventricular wall motion, and severe tricuspid regurgitation (Figure 1). The initial MR and MR angiography of the brain showed no significant abnormalities. A subsequent right heart catheterization at rest confirmed the diagnosis of PAH: the patient had a pulmonary artery pressure of 66/31/43 mmHg, a pulmonary artery wedge pressure of 11 mmHg, a cardiac output of 4.25 L/min and a calculated pulmonary vascular resistance (PVR) of 5.56 Wood units, total pulmonary resistance (TPR) of 7.49 Wood units. Because our patient demonstrated a high pulmonary artery pressure and the cause is unknown. Hence, whole exome sequencing was carried out, and we identified a novel heterozygous mutation in FLNA (c.4754C>T, p.Thr1585Met) and a known heterozygous mutation in MMACHC (c.609G>A, p.Trp203Ter) (Figure 2). In addition, Pedigree analysis of the patient’s son revealed no evidence of these mutations (Figure 2). Following multidisciplinary discussion and being fully informed about the risks to the patient, the patient was initiated on PAH-specific therapy, including tadalafil (20 mg daily), macitentan (10 mg daily), and pulmonary artery denervation (PADN) treatment (Figure 3). Her therapeutic regimens were continued for the next 2 years with periodic follow-up. The timeline of treatment course for this patient was shown in (Table 1). Surprisingly, she showed a satisfactory clinical response to PAH therapies, her disease symptoms were alleviated, and no sign of heart failure was observed during the follow-up period.

**TABLE 1:** The timeline of treatment course for this patient.

Time of admission	Before PAH-targeted therapies	PAH-targeted therapies for 1 year	PAH-targeted therapies for 2 years
LA (cm)	3	3.1	3
LV (cm)	2.7	3.6	3.8
RA (cm)	4.9	4.8	4.7
RV (cm)	4.8	4.6	4.5
EF (%)	53	69	68
TAPSE (cm)	1.4	1.6	1.9
FAC (%)	19	20	22
TR (m/s)	4.8	4.2	3.8
RAP (mmHg)	8	5	3
PAP (mmHg)	62/31/43	53/28/36	46/17/27
RVSP (mmHg)	58	50	47
PCWP (mmHg)	12	10	10
TPR (WU)	7.49	4.31	3.63
PVR (WU)	5.56	3.12	2.27
CO (L/min)	4.25	9.89	10.1
CI (L/min/m^2^)	2.53	5.73	6.5
6MWD (m)	338	370	420
NT-proBNP (pg/mL)	3,358	1,025	336
Functional class (NYHA)	III	II	II
PAH-target therapies	tadalafil (20 mg, qd), macitentan (10 mg, qd), and PADN	tadalafil (20 mg, qd), macitentan (10 mg, qd)	tadalafil (20 mg, qd), macitentan (10 mg, qd)

EF, was measured for the left ventricle; RA, RV, LA, LV, were determined by subxiphoid four chamber section; EF, ejection function; NYHA, New York heart association; NT-proBNP N-terminal pro-brain natriuretic peptide; PAH, pulmonary artery hypertension; RA, right atria; RV, right ventricular; LA, left atria; LV, left ventricular; RVSP, right ventricular systolic pressure; TAPSE, tricuspid annular plane systolic excursion; FAC, fractional area change; RAP, right atrial pressure; PAP, pulmonary arterial pressure; PCWP, pulmonary capillary wedge pressure; TPR, total pulmonary resistance; PVR, pulmonary vascular resistance; CO, cardiac output; CI, cardiac index; 6MWD, 6-min walk distance.

## Discussion

PAH is a fatal cardiovascular disease, often referred to as malignancy of the cardiovascular system. It is a progressive and life-threatening disease driven by the remodeling of peripheral pulmonary arteries, leading to arterial occlusion, increased pulmonary vascular resistance, and elevated mPAP (Grünig et al., 2020). Its hallmark is sustained elevated pulmonary arterial pressure, ultimately leading to right ventricular hypertrophy, heart failure, and eventually death (Eichstaedt et al., 2023). Epidemiological studies have shown that the incidence and prevalence of PAH are 6 and 48–55 cases per million adults, respectively (Leber et al., 2021). The prevalence of PAH is higher in women between the ages of 30 and 60 years (Levine, 2021). The incidence rate among male patients is relatively low, yet their prognosis is often poor. Exercise intolerance is usually the main symptom in those with PAH, seriously affecting their quality of life. Untreated, the median survival is approximately 2.5 years. Although the pathogenesis of pulmonary arterial hypertension is complex, with the development of molecular genetics technology in recent years, the role of gene mutations in PAH has gradually been confirmed, and more and more PAH related genes are becoming well-known. While mutations in *BMPR2* account for the majority of heritable PAH cases, emerging evidence suggests that digenic or oligogenic mutations may contribute to disease severity and phenotypic variability. We are introducing a 47 year old middle-aged female patient who was admitted to the hospital due to long-term chest tightness and shortness of breath. Through genetic testing, it was found that severe pulmonary arterial hypertension was caused by digenic mutations in *FLNA* and *MMACHC*. After standardized drug treatment, the patient’s condition improved significantly.

*FLNA* encodes filamin A, a cytoskeletal protein plays a critical role in cell migration, signaling, and vascular remodeling. *FLNA* gene mutations can cause periventricular nodular heterotopia, which is an X-linked dominant genetic disease in which neurons fail to migrate to the cerebral cortex (Zhou et al., 2021). As well as neurological manifestations, other abnormalities, particularly cardiovascular symptoms and lung diseases, especially pulmonary hypertension, are also common in patients with *FLNA* mutations (Bandaru et al., 2021). Its main clinical manifestations include: bicuspid aortic valve, patent ductus arteriosus, refractory epileptic seizures, intellectual disability, neuronal migration disorders, etc. Previous case reports have also found that a family with heritable PAH carried a novel heterozygous splicing mutation in the *FLNA* gene (Hirashiki et al., 2017). Zheng et al. revealed that *FLNA* knockdown reduced PASMc proliferation and migration, whereas *FLNA* overexpression promoted cell proliferation and migration (Zheng et al., 2023). These indicated that *FLNA* was involved in SMc proliferation and migration, and served a key role in maintaining arterial myogenic tone and vascular remodeling in PAH (Zheng et al., 2023). The novel *FLNA* mutation identified in this patient (c.4754C>T, p.Thr1585Met) likely disrupted protein function, contributing to pulmonary vascular remodeling and PAH progression. Her medical history included intellectual disability, schizophrenia, epilepsy, hearing impairment, and hypothyroidism, which is consistent with the clinical manifestations of *FLNA* gene mutations. *MMACHC* gene deficiency leads to autosomal recessive methylmalonic aciduria combined with hypercystinuria (Zhang et al., 2022). The *MMACHC* gene is located on chromosome 1p34.1, more than 75 mutations have been reported, but the exact function of the *MMACHC* protein is still unclear (Lerner-Ellis et al., 2006). The common clinical manifestations of *MMACHC* gene mutations include developmental delay, intellectual disability, hypotonia, visual impairment, and hematological manifestations. But there are also literature reports that this gene defect may lead to cardiac and pulmonary dysfunction, thereby causing pulmonary arterial hypertension (Hao et al., 2024). Since the discovery of the *MMACHC* gene, mutations have been identified in over 300 patients. The most common genetic abnormality is the c.271dupA, which accounts for more than 40% of mutant alleles (Gündüz et al., 2014). While the *MMACHC* mutation (c.609G>A, p.Trp203Ter) identified in our patient is not classically linked to PAH, its potential role in pulmonary vascular dysfunction warrants further investigation. Our patient exhibits PAH resulting from a heterozygous mutation in two genes. Furthermore, the patient’s favorable response to PAH-specific therapy underscores the importance of early diagnosis and targeted treatment. Genetic screening not only aids in identifying mutation carriers but also provides insights into disease mechanisms, enabling personalized therapeutic approaches. Based on this, we also conducted whole exome sequencing on the patient’s son, but did not identify any carriers of the aforementioned variant genes.

Sympathetic overactivation plays a critical role in elevating PAP. By impairing neural transmission through targeted damage to pulmonary arterial sympathetic nerves, PAP subsequently decreases. This substantial evidence suggests that PADN represents a promising therapeutic approach for pulmonary hypertension. Current indications for PADN treatment are limited to Group 1, 2, and 4 PH according to the World Health Organization (WHO) clinical classification system. Preclinical studies indicated that PADN treatment can significantly reduce mPAP, significantly reduce NTproBNP, significantly improve RV function, and significantly increase 6-MWD (Leopold, 2022). This is consistent with the follow-up results of our patients after treatment. The pulmonary vasculature is densely innervated by sympathetic, parasympathetic, and sensory nerve fibers (Zhang et al., 2022). PAH is associated with hyperactive sympathetic nervous system (Zhang et al., 2022). Excessive activation of the sympathetic nervous system can lead to pulmonary arteriolar vasoconstriction and vascular remodeling. Therefore, PADN treatment for patients with PAH can significantly reduce PAP and improve patient prognosis.

## Limitations and future directions

As a single-case report, our findings require validation in larger cohorts. Multicenter studies screening PAH patients for FLNA/MMACHC mutations are warranted to confirm this digenic association.The mechanistic interplay between FLNA (cytoskeletal remodeling) and MMACHC (metabolic dysfunction) mutations remains speculative. We hypothesize that FLNA-mediated vascular instability may synergize with MMACHC-related mitochondrial dysfunction (impaired energy metabolism in pulmonary artery smooth muscle cells), but this needs experimental verification. PADN was selected over triple therapy due to the patient’s suboptimal response to dual agents (persistent NYHA III after 1 month) and right heart strain on repea echocardiography. While PADN showed efficacy here, its application should be limited to experienced centers until randomized trials establish safety profiles across PAH subtypes.

## Conclusion

This case highlights the rare occurrence of PAH associated with digenic mutations in *FLNA* and *MMACHC*. The patient demonstrated significant clinical improvement following a combination of targeted PAH therapy and PADN, underscoring the therapeutic potential of PADN in managing severe PAH. The reduction in mean mPAP and improvement in right ventricular function observed in this case align with preclinical evidence supporting PADN as a promising intervention for PAH. These findings emphasize the importance of integrating genetic screening with advanced therapeutic strategies, such as PADN, to optimize outcomes in PAH management. Further research is warranted to elucidate the mechanisms underlying PADN’s efficacy and its potential role in broader PAH patient populations.

## Data Availability

The original contributions presented in the study are included in the article/supplementary material, further inquiries can be directed to the corresponding author.
